# Molecular gut content analysis indicates the inter‐ and intra‐guild predation patterns of spiders in conventionally managed vegetable fields

**DOI:** 10.1002/ece3.7772

**Published:** 2021-06-27

**Authors:** Hafiz Sohaib Ahmed Saqib, Pingping Liang, Minsheng You, Geoff M. Gurr

**Affiliations:** ^1^ State Key Laboratory of Ecological Pest Control for Fujian and Taiwan Crops Fujian Agriculture and Forestry University Fuzhou China; ^2^ Joint International Research Laboratory of Ecological Pest Control Ministry of Education Fuzhou China; ^3^ Institute of Applied Ecology Fujian Agriculture and Forestry University Fuzhou China; ^4^ College of the Environment and Ecology Xiamen University Xiamen China; ^5^ Key Laboratory of Integrated Pest Management for Fujian‐Taiwan Crops Ministry of Agriculture Fuzhou China; ^6^ Graham Centre Charles Sturt University Orange NSW Australia

**Keywords:** community assembly, ecosystem services, metabarcoding, niche partitioning, trophic interactions

## Abstract

Inter‐ and intra‐guild interactions are important in the coexistence of predators and their prey, especially in highly disturbed vegetable cropping systems with sporadic food resources. Assessing the dietary range of a predator taxon characterized by diverse foraging behavior using conventional approaches, such as visual observation and conventional molecular approaches for prey detection, has serious logistical problems. In this study, we assessed the prey compositions and compare the dietary spectrum of a functionally diverge group of predators—spiders—to characterize their trophic interactions and assess biological control potential in Brassica vegetable fields. We used high‐throughput sequencing (HTS) and biotic interaction networks to precisely annotate the predation spectrum and highlight the predator–predator and predator–prey interactions. The prey taxa in the gut of all spider families were mainly enriched with insects (including dipterans, coleopterans, orthopterans, hemipterans, and lepidopterans) with lower proportions of arachnids (such as Araneae) along with a wide range of other prey factions. Despite the generalist foraging behavior of spiders, the community structure analysis and interaction networks highlighted the overrepresentation of particular prey taxa in the gut of each spider family, as well as showing the extent of interfamily predation by spiders. Identifying the diverse trophic niche proportions underpins the importance of spiders as predators of pests in highly disturbed agroecosystems. More specifically, combining HTS with advanced ecological community analysis reveals the preferences and biological control potential of particular spider taxa (such as Salticidae against lepidopterans and Pisauridae against dipterans), and so provides a valuable evidence base for targeted conservation biological control efforts in complex trophic networks.

## Graphical abstract

Inter‐ and intra‐guild interactions are important in the coexistence of predators and their prey, especially in highly disturbed vegetable cropping systems with sporadic food resources. In this study, we investigated the trophic interactions of a functionally diverge group of predators—spiders—to accomplish the ultimate goal, that is, the predation of spiders on major crop pests. Despite the generalist foraging behavior of spiders, the community structure analysis and interaction networks highlighted the overrepresentation of particular prey taxa in the gut of each spider family, as well as showed the intrafamily predation between different spiders. Identifying the diverse trophic niche proportions underpins the importance of spiders as predators of pests in highly disturbed agroecosystems.

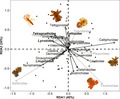

## 1 INTRODUCTION

Understanding trophic interactions among communities is essential to assess how ecosystems function and respond to environmental variations (Michalko et al., 2019; Symondson et al., 2002). Species relationships play a fundamental role in delivering ecosystem services, including the generalist and omnivore predators (Arvidsson et al., 2020; Brechtel et al., 2019; Thébault & Loreau, 2005), for which prey preferences and availability are key (Roubinet et al., 2018; Symondson, 2002). For example, the prey range of a predator depends on its capacity to capture and subdue a given prey type, the influence of competitors, handling time, and availability of alternative prey (Agustí et al., 2003; Friman et al., 2008; Kuusk & Ekbom, 2010; Michalko & Pekár, 2016). Prey choices of generalist predators are potentially highly complex and dynamic in agricultural fields, where densities, diversity, and availability of resources fluctuate temporally and spatially (Cuff et al., 2021; Gurr et al., 2016; Jonsson et al., 2008; Roubinet et al., 2017; Staudacher et al., 2018). Essentially, prey consumption of generalist predators in a given environment largely depends upon what is available and accessible to the predators at any moment. Measuring the predation range of generalists is important for understanding the contribution of each consumer taxon or functional guild to the biological control of pest complexes. However, achieving this understanding by use of conventional approaches such as direct observation of identification of prey fragments in predators’ guts is logistically problematic and time‐consuming (Williams et al., 2012), particularly in the highly dynamic vegetable growing system, and where both prey and predators are small‐sized with short life cycle. Liquid feeders such as spiders present additional challenges.

Notwithstanding the complexities and difficulties of dietary studies, the information from these is valuable in revealing predator–predator and predator–prey trophic interactions, which can be used to underpin efforts to manage and conserve on‐farm biodiversity efficiently, and also help to predict whether a predator group is capable of regulating important ecological processes (i.e., pest suppression) in the field (Amarasekare, 2008; Pompanon et al., 2012). The trophic interactions often require techniques and tools destined for precisely analyzing and depicting the complete dietary spectrum of the generalist predators. In recent years, the development of DNA‐based approaches has considerably improved and encouraged the studies of dietary analysis, and these techniques are widely used to evaluate a range of trophic interactions (King et al., 2008; Pompanon et al., 2012; Symondson, 2002). Conventional DNA‐based approaches can provide accurate information if a predator species consumes an especially important taxon (e.g., diamondback moth in brassicas) or an agonist of such pests (e.g., a key parasitoid) (Agustí et al., 2005; Traugott et al., 2008). However, the classical methods based on prey‐specific primers have become outdated and have limited utility for the dietary analysis of generalist predators and for use in systems where prey ranges are potentially large and not well characterized. The use of multiplexing can enhance the efficiency of this process (Davey et al., 2013; De Barba et al., 2014; Harper et al., 2005; King et al., 2010), but is still limited for the complete dietary analysis.

Recent technical advances and lowering costs for sequencing allow dietary analysis studies to understand the needs of natural enemy species better and predict their role in an ecosystem (Brown et al., 2012). One method, high‐throughput sequencing (HTS) (Sittampalam et al., 1997), enhances the range of prey species detection in the gut or fecal samples. HTS covers the whole prey DNA fragments in the gut of predators, for example, the sequencing of prey species using a DNA barcode or a fragment of mitochondrial cytochrome c oxidase subunit I (COI) gene (Brandon‐Mong et al., 2015; Elbrecht & Leese, 2015; Hamad et al., 2014; Hebert et al., 2003; Mitchell, 2008). HTS technologies provide a more efficient means for untargeted collection of information on the dietary range of predators and prey species (Pompanon et al., 2012).

Spiders are widely distributed in agroecosystems with diverse foraging behavior, so they are expected to be important predators (Arvidsson et al., 2020; Cuff et al., 2021; Mezőfi et al., 2020; Michalko et al., 2019), but the generalist nature of their diets makes it difficult to obtain precise information using conventional approaches, especially given that most spiders are fluid feeders. Further, many spiders are nocturnal hunters, ambush hunters, ground runners, and some hunt away from their webs (Mezőfi et al., 2020; Michalko & Pekár, 2016) making the direct observation of predation events even more difficult. Therefore, there is a great need to develop a precise analytical approach to better understand the trophic niche of hunting spiders using DNA‐based gut content analysis and incorporate these results to highlight their biological control potential. More specifically, our objectives were (a) to determine the composition of prey in the gut and (b) to compare the diet preferences of functionally divergent spider taxa concerning their foraging tactics to elucidate trophic webs and biological control potential in Brassica vegetable fields.

## 2 MATERIAL AND METHODS

### 2.1 Samples collection and identification

Spiders were collected from 17 conventionally managed (i.e., nonorganic) Brassica vegetable fields located in Fujian Province, southeastern China, from August–November in 2017 for one growing season at the time of crop maturity. These sites were mainly covered by the typical conventional Brassica vegetables (mainly including cauliflower and Chinese cabbage crops and fractions of other Brassica crop species) in the autumn season. The different numbers of individuals were collected by randomly searching the plant and soil surfaces for one hour per site within the brassica fields. For molecular gut content analysis, each spider was hand‐collected directly into new clean vials to prevent surface DNA contamination. Vials were immediately transferred to the icebox for transportation to the laboratory and stored at −80°C for future use (see Table S1 for more details of sites). Identifications were performed to family level using a digital microscope, keeping the vials (containing individual spiders) in dry ice during the most identification process to prevent spiders from being unfrozen.

### 2.2 DNA extraction

A total of 156 adult spiders of seven families were used for genomic DNA extractions using DNeasy Blood and Tissue kit (Qiagen Ltd) following the manufacturer's instructions. Individual spiders were surface sterilized with absolute ethanol and washed three times with ddH_2_O. To test the dietary differentiation, spiders of individual families were grouped based on different hunting strategies. Three individuals of the same family collected from conventionally managed fields were pooled to perform a single DNA extraction. A total of 52 DNA extractions were made representing; three of Theridiidae, four of Tetragnathidae, five of Pisauridae, five of Salticidae, seven of Linyphiidae, nine of Lycosidae, and nineteen of Thomisidae. All extracted genomic DNA samples were stored at −80°C till the next use.

### 2.3 PCR amplification and amplicon sequencing

A representative arthropod's universal invertebrate primer pair of COI with barcode primers mlCOlintF as forward (5′‐GGWACWGGWTGAACWGTWTAYCCYCC‐3′) (Leray et al., 2013) and HCO2198 as reverse (5′‐TAAACTTCAGGGTGACCAAAAAATCA‐3′) (Folmer et al., 1994) was used for amplification of a short fragment (~300 bp). The ~300 bp of amplicon sequences located within the COI‐barcode region have been reported to amplify a wide range of invertebrates successfully.

The PCR reaction mixture with a total volume of 50 µl was prepared using Phanta^®^ Max Super‐Fidelity kit (Vazyme Biotech Co., Ltd) contained 3 µl of dNTPs (10 mM each), 25 µl of DNA polymerase buffer, 2 µl of Phanta Max Super‐Fidelity enzyme, 2 µl of each primer (10 µM), 4 µl of DNA template, and final volume adjusted with nuclease‐free water. The PCR thermal cycling conditions were as follows: 95°C for 3 min followed by 16 cycles at 95°C for 30 s, 62°C for 30 s (−1°C /cycle), and 72°C for 60 s, followed in turn by 25 cycles at 95°C for 30 s, 46°C for 30 s, and 72°C for 60 s, and finally 72°C for 300 s. A reaction mixture with no DNA template was used as a negative control in each batch of PCR amplification. 5 µl of each PCR product was used to test the successful amplification of target COI fragment using gel electrophoresis. The remaining PCR product was purified using the PCR Purification Kit (Qiagen). DNA library of successfully amplified samples was generated by pooling of equimolar PCR products and subsequently sent for sequencing on Illunima HiSeq platform according to the manufacturer instruction at Biomarker Inc. (Shanghai).

### 2.4 Bioinformatics

Raw sequencing reads with exact matches to the barcodes were assigned to respective samples and identified as valid sequences. The low‐quality sequences were filtered through the following criteria (Gill et al., 2006; Torondel et al., 2016): sequences that had average QPhred scores of <20, sequences that had a length of <150 bp, sequences that had mononucleotide repeats of >8 bp, and sequences that contained ambiguous bases. Paired‐end reads were assembled using FLASH (Magoč & Salzberg, 2011). A semiautomated bioinformatic channel was generated using Perl to remove the associated tags and primers from each fragment. After chimera detection, the remaining high‐quality sequences were clustered into operational taxonomic units (OTUs) at 97% sequence identity by UCLUST (Edgar, 2010). A representative sequence was selected from each OTU using default parameters. OTUs taxonomic classification was conducted by BLAST (blastn), requiring 98% sequence identity for each representative sequence, blasting the representative sequences against NCBI database (www.ncbi.nlm.nih.gov). An OTU table was further generated to calculate the abundance of each OTU in each sample and the taxonomy of these OTUs. OTUs containing less than 0.001% of total sequences across all samples were discarded.

### 2.5 Statistical data analysis

To determine the prey composition and compare the prey preferences, spiders were pooled at family level. The seven spider families found to be present in the study system had distinct foraging tactics (Linyphiidae = tangleweb‐builder, Lycosidae = active hunter, Pisauridae = roaming hunter, Salticidae = stalkers, Tetragnathidae = orbweb‐builder, Theridiidae = spaceweb‐builder and Thomisidae = ambusher) (Cardoso et al., 2011). Because these families had unique functional traits based on their foraging tactics, we used family as a treatment/predictor in the model. The prey dataset (DNA reads) detected in the gut of spider was used as dependent variable. The “microbiomeSeq” (Torondel et al., 2016) package with its dependencies in R software was used to analyze the alpha diversity (Shannon diversity indices) of prey species in the gut of different spider families. It also measures the pair‐wise ANOVA of diversity indices between groups and generates a box plot for each of the alpha diversity indices interpreted with the level of significance. Before doing further analysis, we performed a relative normalization to the OTU's abundance to obtain the proportion of most abundant prey taxa in the gut of each spider. The local contribution to beta diversity (LCBD) was calculated according to the procedure developed by Legendre and De Cáceres (2013) to measure the level of uniqueness of a given spider to the variations of prey community composition in the gut. Before performing multivariate ordination, Hellinger dissimilarity coefficient method was used to generate the prey community dissimilarities matrices in the gut of spiders since this transformation enables the use of ordination method and gives low weights to variables with low counts and many zeros (Legendre & Gallagher, 2001). Redundancy analysis (RDA) was conducted to better understand the relationship between prey species in the gut with their predators. Multivariate ordination methods (such as RDA) are statistically powerful enough to account for the rare prey species and superdominant prey species at the same time, as well as the differences and similarities among samples can be detected even at much smaller sample sizes (Blanchet et al., 2014; Forcino et al., 2012). The significance of RDA models was tested by performing an ANOVA‐like permutation (999) test (Legendre et al., 2011). Food Web Designer version 3.0 (Sint & Traugott, 2016) was used to quantify the strength of trophic links (proportions of prey DNA reads in the gut) among spiders and different prey groups.

## 3 RESULTS

**FIGURE 1:**
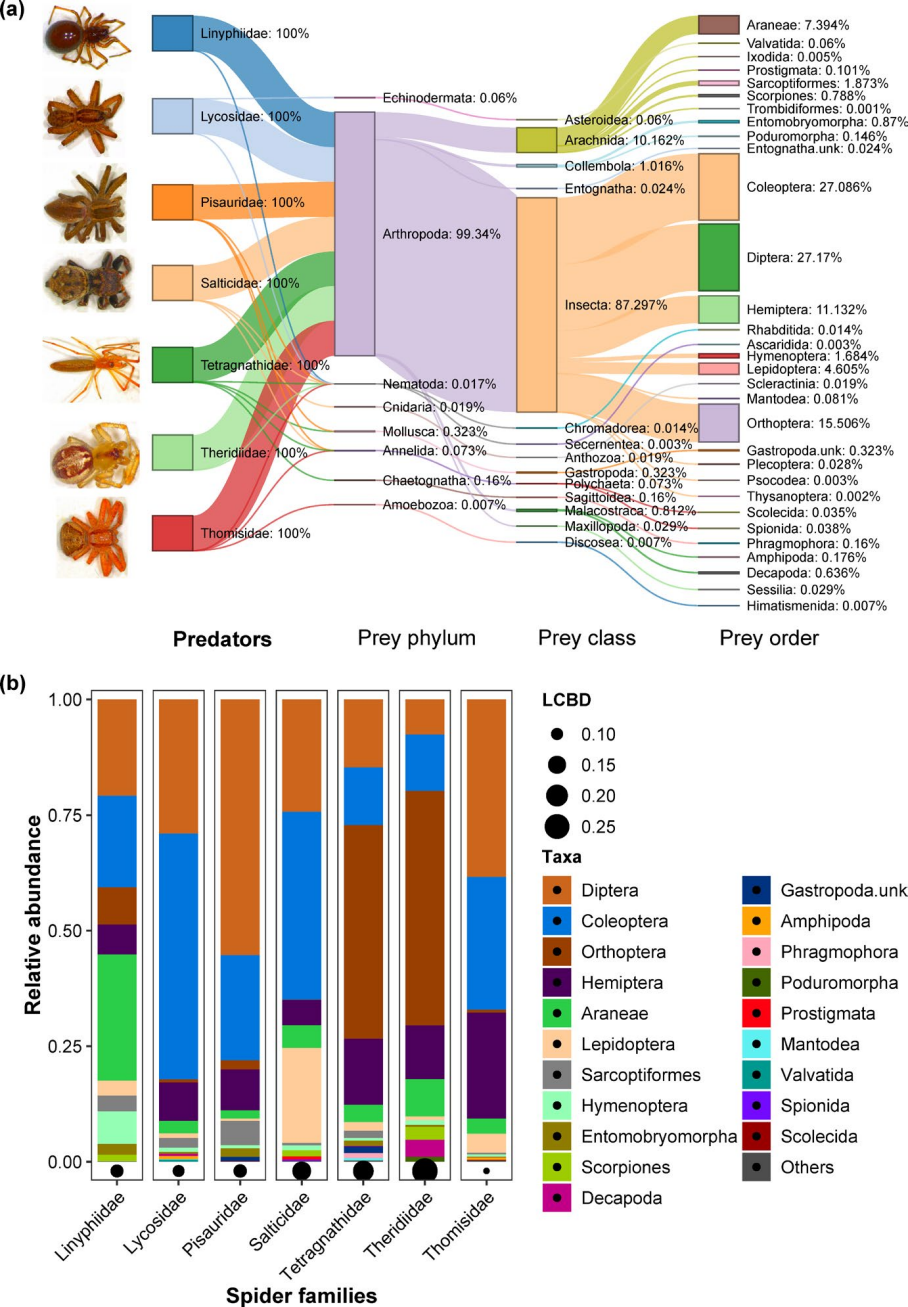
(a) Sankey diagram of proportional abundances of all prey taxa in the gut of different spider families. Arms from left to right denote relative proportions at the phylum, class, and order level of prey groups in the gut of different spider families. (b) Taxa plot represents the 20 most abundant prey orders in the gut of spiders. The local contribution to beta diversity (LCBD) of each spider family showed the beta dissimilarity and uniqueness of prey taxa composition. The plot produced above has black circles at the bottom of each bar; the diameter of the points corresponds to the magnitude of LCBD value

A total of 626,413 clean reads belonging to the 434 unique OTUs were recovered. A total of 66.21% represent the predators (host), arthropods (prey content) share 33.59% of total reads, and only 0.20% belong to other phyla. Among arthropods, the most abundant prey orders were Diptera (27.17%), Coleoptera (27.09%), Orthoptera (15.51%), Hemiptera (11.13%), Araneae (7.39%) (representing the spider families other than the host family), and Lepidoptera (4.61%) detected in the gut of spiders (Figure 1a,b). However, the relative abundance of prey orders between the gut of different spider families was found to be very variable (Figure 1b). The gut of Linipyphiidae had high proportion of predator DNA as compared to the other spider families which had very low proportion of predator DNA than the prey DNA. The LCBD values ranged from 0.10 to 0.25, which are shown for each spider family (Figure 1b), are the comparative index of uniqueness. Large black circles indicate the spider families with strongly different prey species compositions in their gut compared to the other families; these include the Theridiidae, Tetragnathidae, and Salticidae (Figure 1b).

**FIGURE 2:**
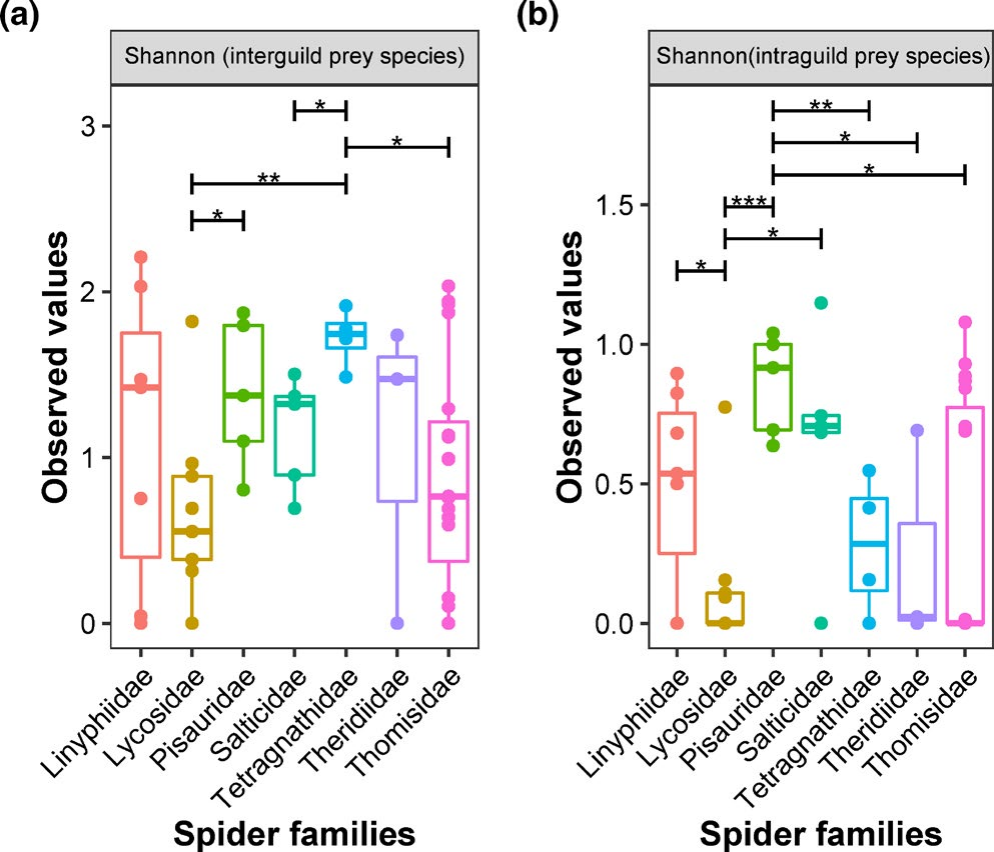
Compare the Shannon diversity indexes with the analysis of variance (ANOVA) for the (a) interguild prey species (other prey species excluding spiders) and (b) intraguild prey species (predation on other spider species) detected in the gut of spiders. Boxplots are drawn, where the box characterizes the interquartile range (25%–75%) and the band inside is the median. Stars “*” represent the level of significance (*p*‐value < .05). Whiskers represent the 1.5 of the lower or upper interquartile range, and outliers are indicated as circles

The highest diversity of prey species (excluding intraguild prey species) was observed in the gut of Tetragnathidae, which was significantly greater (at *p* < 0.05) than the Lycosidae, Salticidae, and Linyphiidae. In contrast, the lowest diversity of prey species was recorded in the gut of Lycosidae, which was significantly lower than the diversity of prey species in the gut of Tetragnathidae and Pisauridae (Figure 2a). Pisauridae had the highest intraguild predation diversity index, which was significantly higher than most of the spider families except Salticidae and Linyphiidae. On the other hand, the gut of Lycosidae showed the lowest intraguild predation diversity, which was significantly lower than the Linyphiidae, Pisauridae, and Salticidae (Figure 2b).

**FIGURE 3:**
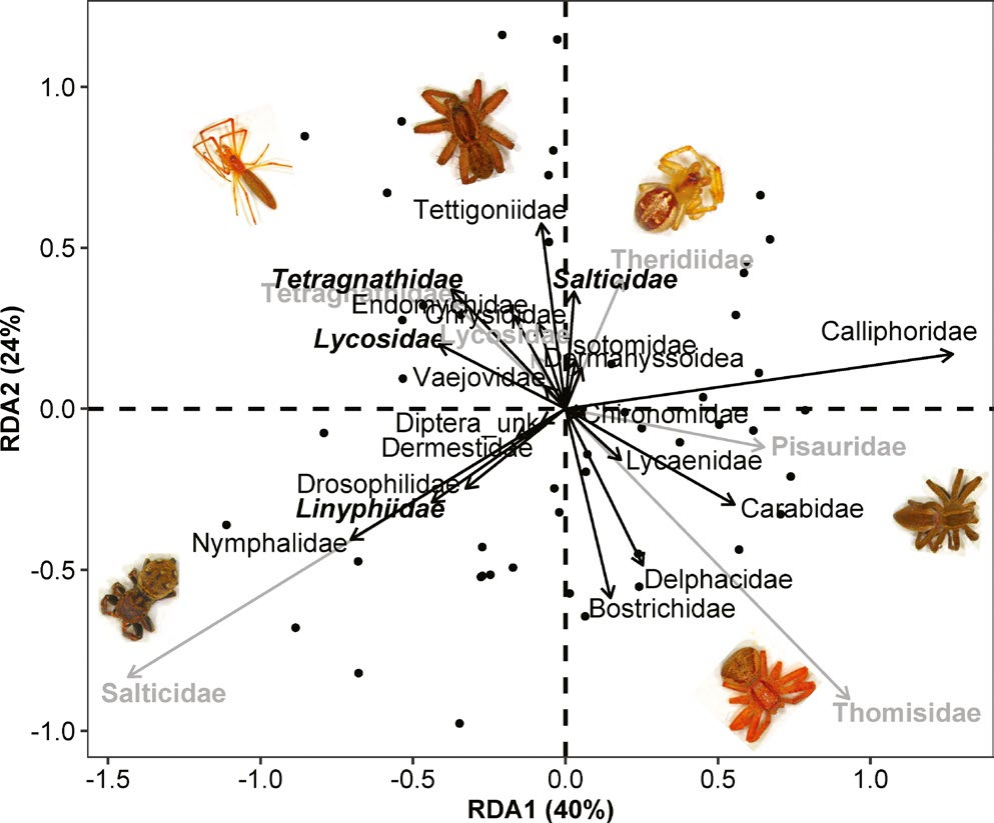
Redundancy analysis (RDA) plot shows the ordination of top 20 prey taxa (text and arrows in black) in the gut of different spider families (bold text and arrows in gray). The arrow length and direction represent the magnitude of variance explained by the explanatory and response variables. The perpendicular distance between spider families and explanatory variables reflects their correlations (below‐90° = positive correlation and above‐90° = negative correlation). The smaller the perpendicular distance, the stronger the correlation. Bold and italic text in black represents those spider families (eaten by other spiders) detected in the gut of host spiders (gray arrows and text)

The differences in profile of gut prey species between different spider families were significant (RDA model permutation test; *F* = 1.396, *p* = 0.043, Figure 3). The results showed that the first two RDA axis accumulatively explained 64% of the total variability in terms of prey species in the gut of different spider families (Figure 3). RDA ordination plot revealed that gut prey species were distinct from each other among different spider families (Figure 3). Tetragnathidae and Lycosidae were clustered together and showed the strongest differences of gut prey species with Salticidae, Pisauridae, and Thomisidae (Figure 3). The trophic patterns of different spider families with diverse foraging strategies are consistent with the conclusion that divergent hunting mode is a strong determinant of gut prey taxonomic composition.

**FIGURE 4:**
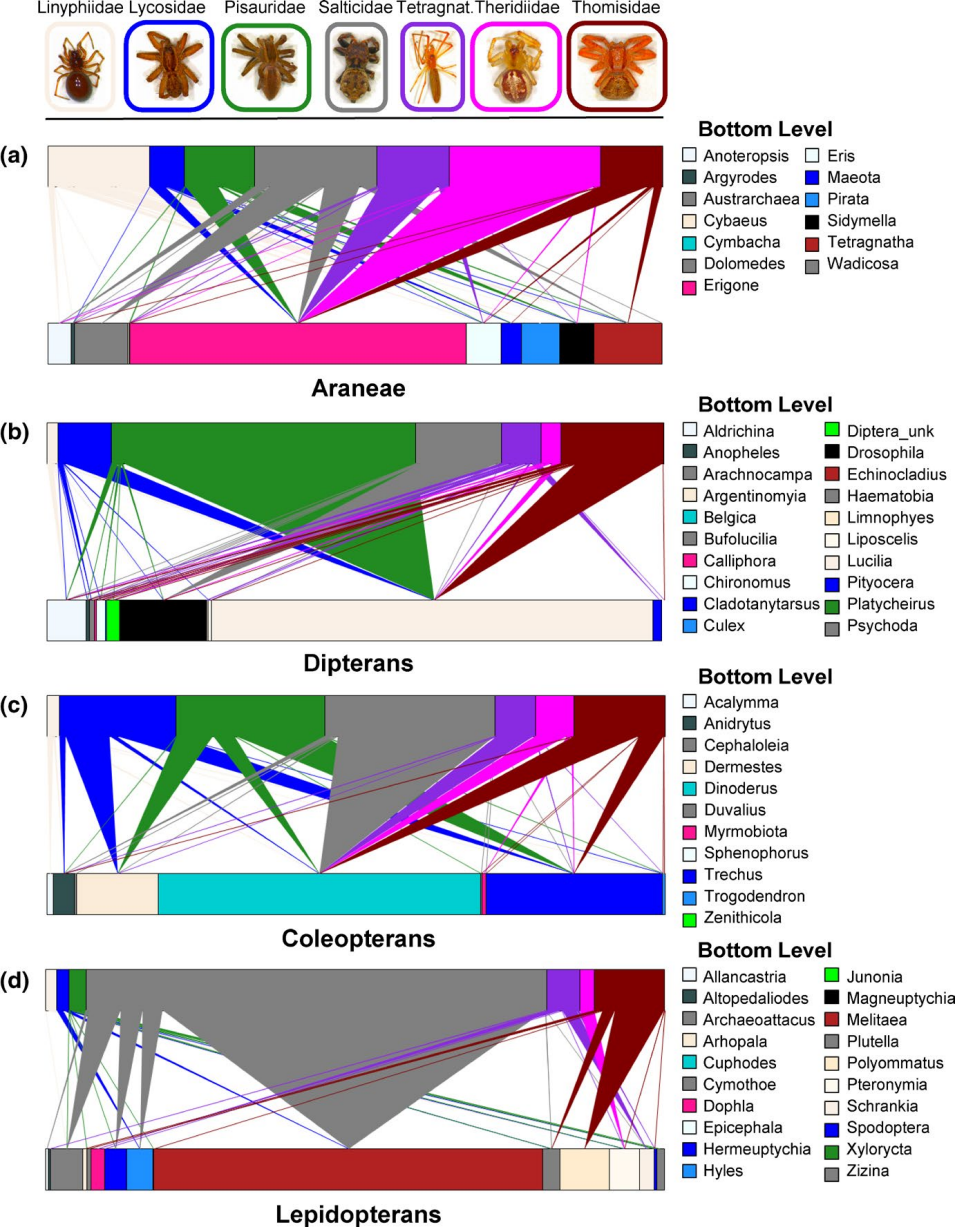
Quantitative trophic networks showing the magnitude of predation on (a) Araneae, (b) dipterans, (c) coleopterans, and (d) lepidopterans prey taxa detected in the gut of different spiders. The top levels represent the spider (higher trophic level), and the bottom level represents the prey taxa. Triangular links between the top and bottom levels show the trophic interactions, with the width of links proportional to the relative percentage of the interaction. For each rectangular bar, its width represents the total number of reads of that respective predator/prey taxon

We detected prey preferences, similarities, and dissimilarities between different spider families when comparing the occurrences of prey species in their gut based on the relative abundance. All spider families had higher predation preference on *Erigone* spp. (Figure 4a). Similarly, highest number of dipterans reads (rectangles in upper level of Figure 4b) were detected in the gut of Pisauridae, followed by Thomisidae, Salticidae, and Lycosidae. Maximum number of Coleopterans reads (rectangles in upper level of Figure 4c) were almost equally shared between of Lycosidae, Pisauridae, Salticidae, and Thomisidae. The gut of all spider families was mainly enriched with *Drosophila* spp. (Figure 4b). The guts of Pisauridae, Salticidae, Tertragnathidae, Theridiidae, and Thomisidae were mainly enriched with *Dinodesrua* spp. except Lycosidae which mainly prey on *Dermestes* spp. and *Trechus* spp. Likewise, Pisauridae and Thomisidae also showed higher predation on *Trechus* spp. (Figure 4c). Salticidae spiders had a relatively higher predation rate on the Lepidoptera (including *Archaeoattacus* spp., *Hermeuptychia* spp., *Hyles* spp., and *Melitaea* spp.). Also, the gut of Thomisidae spiders was mainly enriched with *Plutella* spp. and *Polyommatus* spp., while Tertragnathidae and Theridiidae spiders had an overrepresentation of *Polyommatus* spp. in their guts. Overall, these results clearly highlighted the generalist predation patterns as well as the diet preferences between different spider families. Given over‐ and under‐representations of different prey taxa in the gut of different spider families concluded that differences in the family identity mainly drove these differences.

## 4 DISCUSSION

Mapping the trophic niche breadth of the seven dominant spider families in this highly dynamic ecosystem revealed predation on pest species, as well as the importance of nonpest prey, and strong evidence of inter‐ and intra‐guild predation. In total, 14 classes, 30 orders, and 72 families of invertebrates were detected including the spiders (other than the host family), crickets, flies, cockroaches, beetles, mantids, ants, grasshoppers, mosquitoes, butterflies, wasps, and moths. These results indicated that despite the short growing season of Brassica vegetables and the highly disturbing nature of this cropping system, the diverse spider assemblage consumes a highly diverse and interconnected network of prey, consistent with previous studies suggesting a wide range of prey taxa detected in the gut of spiders (Eitzinger et al., 2019; Kennedy et al., 2019; Zuev et al., 2020).

While spiders as a whole can be characterized as generalist predators (preying on a wide spectrum of prey groups), our results indicated the diet preferences for certain prey taxa in the gut of different spiders. Their divergent ecological and behavioral differences may explain these overrepresentations of certain prey taxa in the diet of different spider families. Additionally, the marked trophic niche differences between different functional clades of spiders, possibly referred to their divergent hunting strategies, such as web‐builders (including Linyphiidae—tangleweb, Tetragnathidae—orbweb and Theridiidae—spaceweb), mostly hunt diurnally by jumping on prey (Salticidae—stalkers), active hunters (Lycosidae—ground runners and Pisauridae—roaming hunters) and ambush hunters (Thomisidae—ambushers). A global‐scale study by Cardoso et al. (2011) indicated that at global levels, spider families are the most practical basis for functional guild classifications. They also suggested that different families with similar guild may present similar roles in an ecosystem. Therefore, even different species in a given functional guild are likely to have similar prey because they shared the same foraging tactic.

Diptera, from 30 prey groups, accounted for 27.17% of all diets, were the largest and most diverse prey groups detected mainly in the gut of Thomisidae (42%) and Pisauridae (32%), followed by Lycosidae (10%) and Salticidae (9%). Similarly, Coleopterans being the second‐largest prey group, accounted for 27% of all diets, were also overrepresented in the diet of Thomisidae (37%), Lycosidae (22%), Salticidae (18%), and Pisauridae (16%). Binford et al. (2016) also reported >50% of *T*. *eurychasma* sampled had dipterans in their jaws. Similarly, another study described that dipterans occupy a very high proportion >75% of the total diet of *T*. *eurychasma* (Kennedy et al., 2019). Dipterans and coleopterans are highly active arthropods and predominantly found in the agricultural fields, facilitating more encounter rates with the predators. Moreover, dipterans may have relatively high nutritional value and did not possess antipredator mechanisms except flight. Overall, the high feeding rate on prey species other than major crop pests (Lepidoptera and Hemiptera) was likely due to the low availability of diamondback moth and cabbage aphid since these are actively targeted by pesticide use. Therefore, spiders will utilize alternate, easily available, and highly nutritional prey in order to maximize their energy uptake to perform several metabolic activities, such as reproduction.

A large number of positive amplifications of small‐sized Linyphiidae (such as *Erigone* spp.) were obtained in the gut of other large‐sized predators correspond well with the feeding competitiveness and intraguild predation of larger and active hunters on smaller and passive hunters (Rypstra & Samu, 2005). However, we could not detect the consumption of intrafamily predation of different spiders, because testing this hypothesis requires more specific primers while only universal metabarcoding primers were used in this study. Despite the limitation associated with the detection of interfamily predation of different spiders, the overrepresentation of small‐sized spiders in the gut of large‐sized spiders may be an indicator of size‐dependent intraguild feeding preferences of different spiders. Overall, the results of this study showed that the gut contents of all spider families had higher proportions of prey DNA than the predator DNA which highlight their importance as biological control of crop pests. Previous studies have also shown that intraguild depends on several factors such prey size, time, and availability of alternate prey. For example, Roubinet et al. (2018) showed that diet of generalist predators is mainly driven by the availability of pest, and detected a higher predation on aphids at mid of cropping season by generalist predators.

To move from the demonstration technology of metabarcoding to an applied, widely employed for monitoring the biodiversity, it must be easy to understand, easy to use, fast, and easy to access. Several studies reported several metabarcoding approaches and technological advancements in developing highly efficient barcode primers, sequencing platforms, and experimental pipelines (Brandon‐Mong et al., 2015; Gomez‐Polo et al., 2016). Our metabarcoding method based on a single set of primers targeting a short fragment of metabarcoding in arthropods has already gained considerable acceptance among researchers studying the conservation of biological control, and it is almost similar to conventional DNA barcoding (Leray et al., 2013). Even though significant progress has been made in metabarcoding during recent years, several issues still need to be resolved to precisely understand the interspecific and intraspecific trophic interactions. The recent use of HTS to characterize the prey communities has revolutionized gut content analysis studies. However, the validity of HTS relies upon several methodological limitations, such as primer efficiency to detect whole prey taxa in the gut of predators (e.g., Pompanon et al., 2012), accurately identifying the prey DNA sequence to a reference database from genetically unclassified areas (A. M. V. Brown et al., 2014; Wilson et al., 2011) and designing methods to detect the intraspecific trophic interactions. The use of advanced HTS techniques to analyze the presence of prey in the gut of spiders permits us to gain a more comprehensive insight into the diversity of invertebrates consumed than conventional molecular gut content approaches and also augmented our knowledge of trophic interactions between foraging strategies of multifunctional taxa of spiders inhabiting the Brassica fields.

In conclusions, we demonstrate strong dietary differences in spider families using the molecular metabarcoding approaches targeting the short fragment of COI, which can offer a broad range of prey detection in the gut of spiders. This methodology could be used to rapidly evaluate anthropogenic effects on biodiversity and ecosystem functioning, particularly in extremely dynamic environments such as vegetables and other annual crops. Overall, this study suggested that trophic preferences and foraging behavior could play a key role in managing the predator and prey species dynamics even in a highly intensive vegetable growing system. Nevertheless, our analysis is based on a single season and conventionally managed fields, so we did not account for the influence of several other key factors involved in driving these trophic interactions such as field management practices, different seasons, cropping patterns, and proportion of different land uses in the surrounding landscape. Therefore, to precisely understand the role of generalists in providing biological control in Brassica fields (characterized by short growing season, high chemical inputs, and high disturbance rate), it is necessary to incorporate the other important predictors of trophic interactions in future studies. Besides, the molecular analysis could reflect the differences in diversity patterns and abundance of prey taxa consumed, and we feel that a molecular analysis protocol integrates with behavioral and metabiological observations would be more promising to offer a broad understanding of the trophic interactions.

## CONFLICT OF INTEREST

The authors declare no conflict of interest.

## AUTHOR CONTRIBUTION

**Hafiz Sohaib Ahmed Saqib:** Conceptualization (lead); Data curation (lead); Formal analysis (lead); Investigation (lead); Methodology (lead); Software (lead); Validation (lead); Visualization (lead); Writing‐original draft (lead); Writing‐review & editing (lead). **Pingping Liang:** Formal analysis (supporting); Software (supporting); Writing‐review & editing (supporting). **Minsheng You:** Conceptualization (lead); Funding acquisition (lead); Project administration (lead); Resources (lead); Supervision (lead); Writing‐review & editing (lead). **Geoff** **Gurr:** Conceptualization (lead); Funding acquisition (lead); Project administration (lead); Resources (lead); Supervision (lead); Validation (lead); Writing‐original draft (lead); Writing‐review & editing (lead).

## Supporting information

Table S1

## Data Availability

Raw sequence data have been deposited in the Dryad digital repository (https://doi.org/10.5061/dryad.2bvq83bph).
