# Supplementary material for: Molecular gut content analysis indicates the inter‐ and intra‐guild predation patterns of spiders in conventionally managed vegetable fields
Source: Ecol Evol. 2021 Jun 27;11(14):9543–52. doi: 10.1002/ece3.7772 (PMC8293772; doi:10.1002/ece3.7772)
Supplement: Supplementary file 1 — Table S1 [file ECE3-11-9543-s001.docx]

Supplementary information

Molecular gut content analysis indicates the inter- and intra-guild predation patterns of spiders in conventionally managed vegetable fields

Hafiz Sohaib Ahmed Saqib ^1,2,3^, Pingping Liang ^4^, Minsheng You ^1,2,3,5,^* and Geoff M. Gurr ^1,2,3,6,^*

^1^ State Key Laboratory of Ecological Pest Control for Fujian and Taiwan Crops, Fujian

Agriculture and Forestry University, Fuzhou 350002, China

^2^ Joint International Research Laboratory of Ecological Pest Control, Ministry of

Education, Fuzhou 350002, China

^3^ Institute of Applied Ecology, Fujian Agriculture and Forestry University, Fuzhou

350002, China

^4^ College of the Environment and Ecology, Xiamen University, Xiamen 361102, China

^5^ Key Laboratory of Integrated Pest Management for Fujian-Taiwan Crops, Ministry of

Agriculture, Fuzhou 350002, China

^6^ Graham Centre, Charles Sturt University, Orange, NSW 2800, Australia

* Corresponding authors e-mail: [msyou@fafu.edu.cn](mailto:msyou@fafu.edu.cn); [ggurr@csu.edu.au](mailto:ggurr@csu.edu.au)

**Table S1** Information corresponds to the conventionally managed Brassica field locations to collect the spiders for gut content analysis.

| **Sample** | **Latitude** | **Longitude** |
| --- | --- | --- |
| Baizhong | 26° 7' 53.81" | 118° 43' 49.11" |
| Bandong | 26° 7' 46.36" | 118° 44' 35.25" |
| Nantong4 | 25° 55' 29.06" | 119° 15' 43.73" |
| Rongyuan | 26° 15' 19.44" | 119° 5' 41.13" |
| Xiqian | 26° 09' 56.52" | 118° 46' 19.46" |
| Guanzhuang | 26° 09' 47.02" | 118° 50' 11.06" |
| Xinan | 26° 10' 48.51" | 118° 47' 26.48" |
| Dahuxiang2 | 26° 20' 47.02" | 119° 05' 37.27" |
| Dahuxiang1 | 26° 20' 50.1" | 119° 05' 00.45" |
| Dahuxiang3 | 26° 20' 15.14" | 119° 05' 33.72" |
| Nantong1 | 25° 57' 31.20" | 119° 15' 19.25" |
| Nantong3 | 25° 55' 56.14" | 119° 15' 24.96" |
| Jiantian | 26° 07' 47.62" | 119° 19' 48.20" |
| Nantong2 | 25° 56' 40.61" | 119° 15' 23.17" |
| Chengmen | 25° 59' 44.56" | 119° 24' 08.87" |
| Pudang | 26° 07' 50.51" | 119° 19' 56.55" |
| MinqingBA | 26° 13' 41.96" | 118° 51' 21.85" |
